# A method for improving SELDI-TOF mass spectrometry data quality

**DOI:** 10.1186/1477-5956-5-14

**Published:** 2007-09-05

**Authors:** Toni Whistler, Dominique Rollin, Suzanne D Vernon

**Affiliations:** 1 Chronic Viral Diseases Branch, Centers for Disease Control and Prevention, 1600 Clifton Rd, G41, Atlanta, Georgia, 30329, USA

## Abstract

**Background:**

Surface-enhanced laser desorption/ionization time-of-flight mass spectrometry (SELDI-TOF MS) is a powerful tool for rapidly generating high-throughput protein profiles from a large number of samples. However, the events that occur between the first and last sample run are likely to introduce technical variation in the results.

**Methods:**

We fractionated and analyzed quality control and investigational serum samples on 3 Protein Chips and used statistical methods to identify poor-quality spectra and to identify and reduce technical variation.

**Results:**

Using diagnostic plots, we were able to visually depict all spectra and to identify and remove those that were of poor quality. We detected a technical variation associated with when the samples were run (referred to as batch effect) and corrected for this variation using analysis of variance. These corrections increased the number of peaks that were reproducibly detected.

**Conclusion:**

By removing poor-quality, outlier spectra, we were able to increase peak detection, and by reducing the variance introduced when samples are processed and analyzed in batches, we were able to increase the reproducibility of peak detection.

## Background

Surface-enhanced laser desorption/ionization time-of-flight mass spectrometry (SELDI-TOF MS) allows users to generate protein expression data rapidly from a large number of samples and has been used increasingly to identify diagnostic biomarkers of cancer [1-3], mental illness [4,5], and neurological disorders [6,7]. However, as with any analytic technique, its results must be reproducible if one is to have confidence in them.

Several challenges to implementing SELDI-TOF MS in routine clinical diagnostics have already been overcome [8-10]. These include challenges pertaining to biologic samples such as the characterization of sample donors (e.g., by age, sex, fasting status, diurnal rhythm) [11]; sample collection and handling [12,13]; and the effects of freezing, thawing, and storage on specimen stability [14]. Parameters of the SELDI-TOF MS technique that have been assessed range from its sample-processing and robotic-handling systems to its application of the energy-absorbing matrix [15-17]. Finally, many aspects of the technique designed to improve the calibration and quality of the spectra [10,18-21] and of peak detection and quantification [22-24] have made SELDI-TOF MS one of the most promising protein biomarker discovery methods.

Even though a variety of software packages can be used to analyze SELDI-TOF MS data, few are effective in averaging replicate spectra or identifying poor-quality spectra [25,26], and none are capable of analyzing and adjusting for the variation introduced when samples are processed and analyzed in batches. We demonstrate that conventional statistical approaches can be used to identify outlying spectra and correct for batch variation, as well as to increase the number of peaks detected by SELDI-TOF MS and improve the reproducibility of peak detection.

## Results

**Figure 1:**
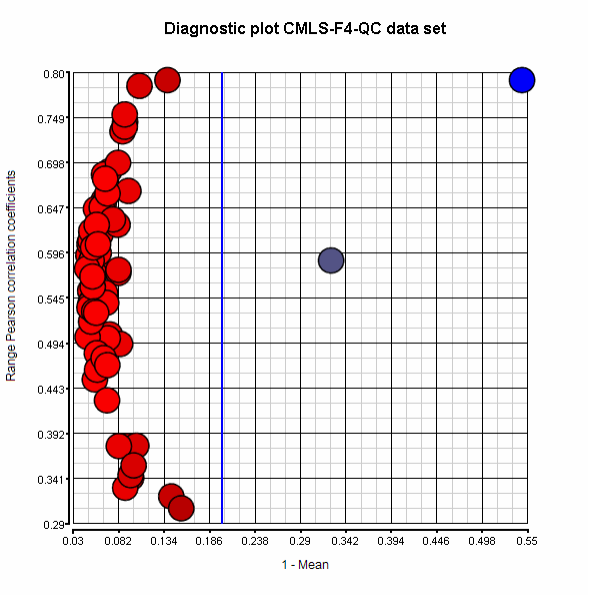
**Diagnostic plot generated from a Pearson correlation matrix of 66 QC spectra from the CMLS-F4 data set**. A cut-off value of 1-Mean of Pearson correlation coefficient > 0.2 was used to exclude spectra from the QC analysis (blue line). In this data set, 2 QC sample spectra were above this cut-off (colored blue) and therefore were removed prior to further processing. For spectra from the investigational data set, a 1-Mean > 0.4 was used for all ProteinChip-Fraction combinations.

To identify and remove poor-quality spectra, we assessed the degree of linear relationship among all spectra in each data set (a ProteinChip-fraction combination). We then generated a pair-wise similarity matrix using the Pearson correlation coefficient on normalized intensity values of each spectrum. To visually depict the data, we drew a diagnostic plot of 1 minus the mean (1-mean) of Pearson correlation coefficients (x-axis) against the range of correlation coefficients (y-axis) (Figure 1). By comparing the results depicted in these diagnostic plots to other evaluation methodologies, such as principal component analysis of the processed spectra or signal to noise (SN) ratios, and by comparing the number of peaks in each spectrum with the average number of peaks for all spectra in the data set, we established cut-off values of 1-mean > 0.2 for QC spectra and > 0.4 for specimen spectra.

**Figure 2:**
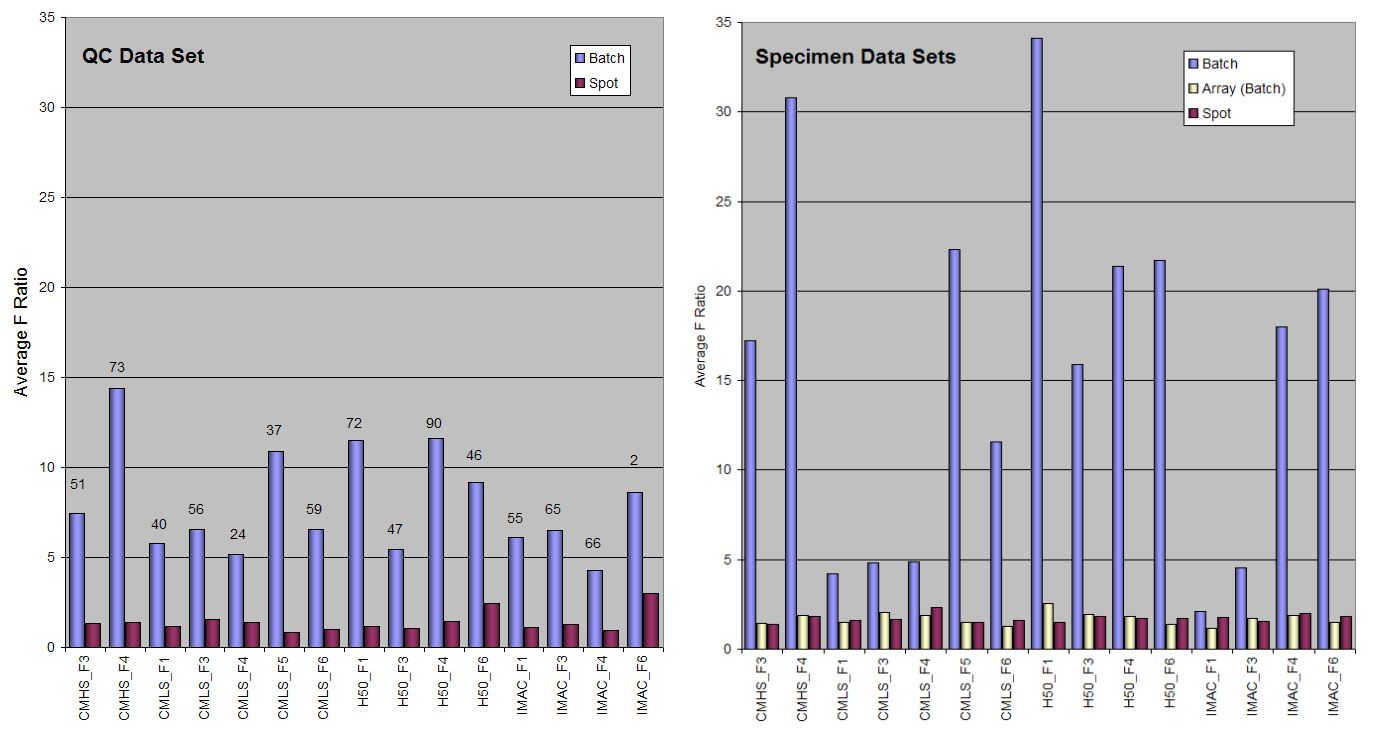
**Sources of technical variation for the QC (left) and Investigational (right) data sets prior to the Partek Batch Removal process**. A plot of the average F ratio (the signal-to-noise ratio) produced when we applied the ANOVA model (Batch and Spot) to the QC data sets (left), and the Investigational data set (right) which also included ProteinChip lot number (Array). Analysis was performed on spectra after removal of outlier spectra, prior to technical effect batch removal. Batch effect is the largest contributor to variance. The numbers above each group indicate the percentage of peaks that were different in at least one batch (*p *> 0.005) as determined by the Kruskal-Wallis test. The QC data set is derived from a pooled serum sample so no. After batch removal, none of the peaks were significantly different.

Variation in analytic results is introduced when samples are processed and analyzed in different batches. To examine the extent of this batch effect, we used the nonparametric Kruskal-Wallis test to compare the normalized peak intensities in the spectra within a batch to the same peak (by mass-to-charge (m/z) value) in the spectra from all other batches. Our null hypothesis was that intensity means would be identical for each peak across the different batches. Using a corrected *p*-value of < 0.005 to calculate the number of peaks that were different in at least one batch, we found a statistically significant batch effect in at least 50% of peaks for each ProteinChip-fraction combination (Figure 2).

We used a 2-way analysis of variation (ANOVA) model to explore batch effect variation in the QC sample and a 3-way ANOVA model to explore batch effect variation in the investigational samples. The batch from which spectra were processed was the largest source of variation in both the QC and the investigational samples (Figure 2). The range of the F ratio (or signal-to- noise ratio) was 4 to 14 for the QC sample, much lower than the 2 to 34 F ratio range for the investigational samples. The CM10 low-stringency (CMLS) fractions 1 to 4 (F1-F4) and the IMAC F1 and F3 ProteinChips showed the lowest batch variance, with QC and investigational samples having similar F ratios (Figure 2).

**Figure 3:**
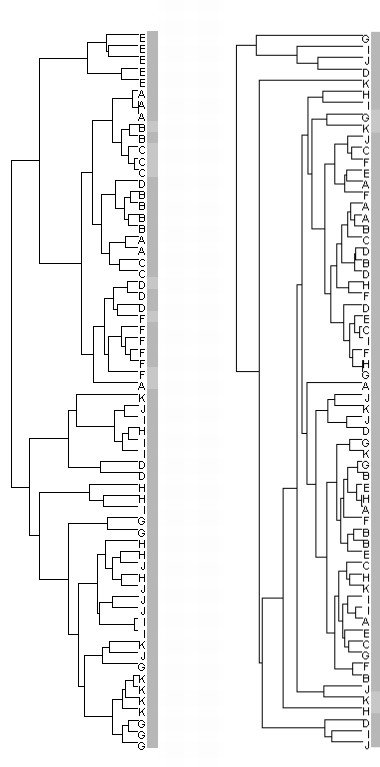
**Dendrograms from hierarchical cluster analysis of spectra from CMLS-F4-QC data set labelled by batch, processed before (left) and after (right) Partek Batch Removal**. QC spectra peak intensities from different points in the analytical process were used to generate dendrograms from hierarchical cluster analysis (Spearman rank dissimilarity metric with average linkage). Each spectrum was labeled for the batch in which it was processed (A through K). The dendrogram on the left is from analysis of spectra before Partek Batch Remover was applied to the data set. Spectra cluster in nodes according to the batch in which they were processed. Two large clusters are evident. One with spectra from batches A through F. The second covering batches G through K. An unanticipated instrument adjustment had to be made between the sixth and seventh batch, which is noticed in the analysis. The dendrogram on the right shows the hierarchical cluster analysis of the same data after Partek Batch Remover was used to reduce the contribution of batch effect technical variance. Spectra no longer cluster by batch in which they were processed, and spectra from before and after the instrument maintenance are inter-mingled across the 2 major nodes.

As described in the Methods section, we used the Batch Remover tool (Partek Genomics Suite) to reduce the effects of batch variation. Hierarchical clustering of spectra in each data set showed that before we used the Batch Removal tool, each batch clustered as a distinct node (Figure 3 for CMLS-F4-QC). In fact, 2 nodes were apparent in all data sets, one for batches run prior to unexpected instrument maintenance being done (batches A through F) and one for batches run after such maintenance was done (batches G through K). After we applied the Batch Removal tool, however, we observed no clustering by batch (Figure 3).

We assessed the quality of each spectrum by using the Pearson correlation coefficient to compare the 11,876 intensity measures of each spectrum. Using the cut-off criteria we established of 1-mean > 0.2 for QC spectra and > 0.4 for specimen spectra, we obtained very similar results if we used peak intensities (less than 100 values per spectrum) to generate the correlation matrix. Before the outlier spectra and batch effect variance were removed, the correlation coefficients ranged from 0.75 to 0.95 in each full data set (Table 1A). Removing poor-quality spectra improved the correlations, 0.88 to 0.96 (Table 1B) as did removing the batch effect, 0.95 to 0.99 (Table 1C). Duplicate spectra from individual samples showed a high degree of reproducibility as demonstrated by a median Pearson correlation coefficient of 0.98 for the 207 pairs of spectra in the CMLS-F4 data set. Results for the other data sets were similar (results not shown).

**Table 1:** Summary data showing stages in the quality assessment of QC sample spectra.

Analysis Step	QC Data Set	Pearson Correlation Coefficient (Grand Mean ± Std Dev)	No. spectra	No peaks	Coefficient of Variation (%)			
					
					Low	High	Average	% Peaks CV < 30%
A	CMHS_F3	0.834 ± 0.225	66	58	15.1	199.2	44.8	34
	CMHS_F4	0.829 ± 0.135	66	37	14.1	84.7	37.9	27
	CMLS_F1	0.946 ± 0.038	66	68	8.0	48.1	22.5	82
	CMLS_F3	0.851 ± 0.142	66	72	7.8	50.1	26.3	69
	CMLS_F4	0.918 ± 0.110	66	54	9.8	53.7	24.1	76
	CMLS_F5	0.879 ± 0.146	66	54	10.5	274.4	32.9	70
	CMLS_F6	0.904 ± 0.173	66	57	9.2	47.6	26.4	61
	H50_F1	0.948 ± 0.065	66	35	17.4	125.1	45.6	37
	H50_F3	0.844 ± 0.189	66	78	8.3	233.0	28.6	67
	H50_F4	0.891 ± 0.084	66	69	11.8	110.7	28.9	71
	H50_F6	0.790 ± 0.256	66	47	16.9	155.5	43.9	36
	IMAC_F1	0.939 ± 0.038	66	66	9.5	54.8	29.9	59
	IMAC_F3	0.884 ± 0.112	66	57	13.1	65.6	34.1	39
	IMAC_F4	0.911 ± 0.080	66	50	14.3	48.3	27.4	68
	IMAC_F6	0.728 ± 0.265	66	55	9.7	66.8	35.4	36

B	CMHS_F3	0.940 ± 0.037	58	53	9.8	86.0	33.2	57
	CMHS_F4	0.878 ± 0.085	59	41	12.1	89.1	34.7	44
	CMLS_F1	0.946 ± 0.038	66	68	8.0	48.1	22.5	82
	CMLS_F3	0.911 ± 0.059	57	75	7.2	45.4	23.9	77
	CMLS_F4	0.941 ± 0.049	64	55	9.7	41.8	21.9	84
	CMLS_F5	0.910 ± 0.060	63	52	10.4	47.8	24.2	77
	CMLS_F6	0.935 ± 0.055	65	71	9.2	50.6	24.6	69
	H50_F1	0.961 ± 0.037	65	83	14.3	131.2	49.4	24
	H50_F3	0.922 ± 0.055	58	78	8.2	65.3	21.6	82
	H50_F4	0.905 ± 0.068	63	69	8.5	111.8	26.9	74
	H50_F6	0.922 ± 0.057	48	46	14.8	169.1	36.0	43
	IMAC_F1	0.939 ± 0.038	66	66	9.5	54.8	29.9	59
	IMAC_F3	0.925 ± 0.045	61	60	12.5	58.3	31.8	43
	IMAC_F4	0.921 ± 0.061	65	50	14.2	47.4	26.9	68
	IMAC_F6	0.929 ± 0.041	16	60	7.0	46.2	23.6	70

C	CMHS_F3	0.980 ± 0.018	58	53	8.1	50.4	19.1	92
	CMHS_F4	0.971 ± 0.027	59	41	5.5	24.6	16.2	100
	CMLS_F1	0.975 ± 0.026	66	68	5.3	30.0	15.1	100
	CMLS_F3	0.961 ± 0.052	57	75	5.2	25.7	13.9	100
	CMLS_F4	0.972 ± 0.039	64	55	5.9	26.2	14.4	100
	CMLS_F5	0.979 ± 0.018	63	52	5.8	30.8	13.0	98
	CMLS_F6	0.975 ± 0.024	65	71	5.2	42.3	15.8	96
	H50_F1	0.990 ± 0.012	65	83	10.6	81.3	26.5	72
	H50_F3	0.982 ± 0.012	58	78	5.8	40.4	13.5	95
	H50_F4	0.977 ± 0.019	63	69	5.0	50.9	13.7	94
	H50_F6	0.971 ± 0.028	48	46	7.1	51.8	17.1	91
	IMAC_F1	0.976 ± 0.197	66	66	7.1	43.2	19.5	92
	IMAC_F3	0.968 ± 0.045	61	60	8.6	33.5	19.3	95
	IMAC_F4	0.945 ± 0.055	65	50	10.2	39.7	20.8	88
	IMAC_F6	0.989 ± 0.009	16	60	2.5	29.0	7.7	100

Pearson Correlation Coefficients were calculated on the entire spectrum prior to peak detection, the coefficient for the entire dataset is reported (Grand statistic). The coefficient of variation (CV) is calculated on all peak intensities.

(A) All spectra in each QC sample data sets

(B) Spectra remaining after passing diagnostic plot cut-off of 1-Mean r < 0.2

(C) Spectra after Partek Batch Removal.

To measure the reproducibility, we calculated the coefficient of variation for the peak intensities of all spectra in each QC sample data set (Table 1). Similar data is available for the investigational samples [see Additional file 1]. The removal of low-quality spectra generally improved the number of peaks common to all spectra in that data set and reduced the average CV for the full spectrum (Table 1B). Batch removal produced a more dramatic effect (Table 1C): the number of peaks remained the same, but the average CV improved as did the number of peaks in each data set with a CV < 30% (Table 1). For example, the CMLS_F5 QC serum data set started with 66 spectra with 54 peaks present; the average CV for specimens in this set was 70% (range: 11–274%, Table 1A). Using the diagnostic plot criteria, we removed two spectra, thereby reducing the CV range to 10–48% and the average CV to 24% (Table 1B). Removing the batch effect technical variance further reduced the CV range to 6–31% and the average CV to 13% (Table 1C). We obtained similar results with the specimen data sets [see Additional file 1]. For all data sets, the CV for m/z values were within the 0.3% reported in the literature [19].

## Discussion

Even though SELDI-TOF MS is designed as a high-throughput automated assay, large studies involving many biological samples are often divided into batches that are analyzed over several days to weeks. To detect any variability that may occur, analysts process pooled human serum (QC samples) with the study samples. In this study, we used an ANOVA model to assess technical variance in peak intensities that could be introduced by differences among sample batches, variations in the spot position of each sample on the ProteinChip, and variations in the ProteinChip array. We found that batch differences accounted for the largest source of technical variability in each data set, with variations in spot position and ProteinChip array contributing little. Therefore, any analysis that ignores the variation associated with processing samples in different batches leaves a considerable amount of noise in the data. The balanced design of the experiments we conducted allowed us to reliably estimate the batch effect and then to remove that effect using the Partek Batch Remover (based on a mixed-model ANOVA). As only technical factors were included in the ANOVA model, the peak intensity data can then be used in further statistical analyses.

Hong *et al*. [27] identified the correlation matrix as an effective metric for identifying lower quality spectra. However, we found that this approach was less effective when used to establish one cut-off value for several large data sets. In an attempt to automate our decisions on which spectra should be included in our analysis, we drew on our knowledge of microarrays and presented our data in diagnostic plots [28] (Figure 1). The results we obtained using diagnostic plots to assess poor spectrum quality compared favourably with assessments based on visual inspection and normalization factors >2 standard deviations from the mean (as recommended by Ciphergen Biosystems, Freemont, USA). Our use of statistical measures to assess spectrum quality allowed us to automatically remove even more poor-quality spectra. There are other measures that could be considered in determining data quality, for example peak resolution [18,19], however the software packages used in this analysis did not determine this parameter.

QC data sets represent the same pooled serum sample run with each batch of investigational sera. This directly evaluates the repeatability of measurements and a more stringent cut off value (1-mean > 0.2) is used with the QC data sets compared to the investigational data sets. The repeatability is expected to be much higher in the QC data sets. Good performance should be associated with low coefficients of variation for the peak intensities, as the data are all derived from the same pooled reference serum. Table 1 illustrates the improvements in data quality and reproducibility resulting from the removal of outlying, poor-quality spectra and the removal of the technical batch effect. The average CVs for all data sets (except H50-F1) were ≤ 20% when all peaks were considered rather than just 3 to 7 major peaks as reported in some studies [29,30]; furthermore, more than 90% of all peaks in each data set, other than H50-F1, had peak intensity CVs < 30%.

## Conclusion

In this study, we used a diagnostic plot to detect and discard low-quality spectra. This method was easy to implement and effective in detecting outlier spectra. Our use of the model-based ANOVA to account for the technical variance introduced by batch processing of spectra further improved the data quality.

## Methods

### Samples

A reference or QC sample was prepared by pooling serum collected in Vacutainer tubes with no additives from 10 donors. This was processed, aliquoted and frozen in the same manner as study subject samples. Serum samples from 207 subjects (referred to as investigational sera) were collected during a clinical study of Chronic Fatigue Syndrome in Wichita Kansas [31].

### Serum fractionation

All of the experimental protocols were performed by a single laboratorian.

To reduce sample complexity and increase the number of protein peaks detected, we performed anion exchange fractionation using the Expression Difference Mapping™ Kit – Serum Fractionation (Ciphergen Biosystems Inc., Fremont, CA, USA) the robotic Biomek 2000 liquid handling system (Beckman Coulter, Fullerton, CA, USA). We collected six different fractions – pH 9 (F1), pH 7 (F2), pH 5 (F3), pH 4 (F4), pH 3 (F5) and organic (F6) – from investigational serum samples that were fractionated in 11 batches over a period of 7 months. Twenty investigational samples and 3 QC samples were processed in each batch and then frozen at -80°C. For each batch, we analyzed fractions in the same order and kept freezing times (2 to 11 days) and processing conditions constant.

### Protein expression profiling

Aliquots of each fraction were bound in duplicate with a randomized ProteinChip/spot position allocation scheme to 3 different types of ProteinChip arrays: IMAC-Cu (metal binding), H50 (hydrophobic chemistry) and two CM10 (anionic chemistry) ProteinChip arrays. One for a high stringency (HS) wash using 50 mM HEPES, pH7 performed before sample application to allow selective binding of proteins, and one for low stringency (LS) wash, 0.1 M sodium acetate pH4, performed before sample application. From previous studies [32], we know that F2 is not particularly informative, and F5 has many overlapping peaks present in F4 and/or F6. Therefore, we did not run these fractions in this study.

For each ProteinChip array, the relevant QC fraction was present on one spot position. The details of ProteinChip processing have been described previously [32]. We used saturated sinapinic acid in 50% acetonitrile/0.5% trifluoroacetic acid as matrix and applied it using the robot. We read the ProteinChips in a PBSIIc mass spectrometer (Ciphergen Biosystems) using automated data collection protocols with previously optimized conditions [32]. We used data from the low mass range protocols (3000 to 30,000 Daltons) in our analysis and calibrated for mass accuracy using the "all-in-one" protein standard II on NP20 ProteinChips (Ciphergen Biosystems). The "all-in-one" peptide standard should be used if a greater accuracy is required at m/z < 8,000 applied with the sinapinic acid matrix to keep data comparable.

Instrument performance and evaluation are critical to spectrometer function and complete details of calibration, alignment and accuracy assessments performed routinely are fully outlined in a previous publication [32].

Using data from 15 fractions, we generated 414 investigational spectra and 66 QC spectra per ProteinChip-fraction, each of which we considered a data set. We had to make an unanticipated instrument adjustment, which involved a preventative maintenance service, between the sixth and seventh batches because of laboratory relocation.

### Processing of spectral data sets

We used the QC serum sample to develop and evaluate data processing procedures, which we then used in processing data for the 207 investigational samples.

We exported raw spectrum data files for each ProteinChip-fraction and processed them using the following calibration equation:

*mz *= *U*(*a*(*t *- *t*_0_)^2 ^+ *b*)

Where m/z is the mass-to-charge ratio, U is the voltage (20,000 for this data set), and t is the time-of-flight. For our mass calibration, we used the values, a = 0.336302, b = 0, and t_0 _= 0.09, which we obtained from the calibration equation generated from the protein standard. The final spectrum, from m/z 3,000 to 30,000, generated 11,876 data points. We saved the m/z and intensity values as comma-separated values files. We used SpecAlign [33] to pre-process each spectral data set of QC spectra (66 per ProteinChip-fraction) and specimen spectra (414 spectra per ProteinChip-fraction). We then followed the steps below to process the data:

1. Smooth the data using the Savitzky-Golay filter with a setting of 8.

2. Denoise the spectra using a wavelet transform with a threshold setting of 0.5.

3. View baseline subtraction using a window setting of 5.

4. Subtract baseline.

5. Rescale intensity values to positive.

6. Normalize intensity values using Total Ion Current.

7. Generate an average spectrum.

8. Align spectra using the combined Fast Fourier Transform (FFT)/Peak matching method on the full m/z range, with a scale of 1, a maximum shift of 20, looking ahead by 1, and using the average as a reference.

9. Export the processed data as a single file (to be used for correlation analysis).

10. Pick peaks with a baseline cut-off of 0.5, a window of 10, and a height ratio of 1.5

11. Export peak intensity values for all spectra in a single file.

### Statistical Analysis

We performed all statistical analysis using Partek Genomics Suite software, version 6.2 (Partek Inc., St. Charles, Missouri).

To detect outlier spectra, we used full spectrum processed data consisting of 11,876 intensity values covering the m/z range from 3,000 to 30,000 (the data file exported in step 9 above). We also generated a similarity matrix using the Pearson correlation coefficient on all combinations of spectra within the data set. We then calculated a mean correlation coefficient for each spectrum and visually depicted the coefficients on diagnostic plots [28]. Our cut-off criteria were 1-mean > 0.2 for the removal of QC spectra and > 0.4 for the removal of spectra from investigational samples.

After the quality assessment of the spectra and prior to the batch removal process, we used a 2-way ANOVA model to determine the variation in the data sets. Variation in the QC data sets attributable to the batch process (Batch, Figure 2) and to the position on the ProteinChips (Spot, Figure 2) was evaluated. For the larger investigational data sets, we used a 3-way model that also incorporated ProteinChip array (Array, determined by Lot Number) as a factor (nested in Batch). The Partek Batch Remover that we used employs a mixed-model ANOVA of the technical factors to identify and remove these sources of variation. The variation is reported as the average F ratio, a measure of the average signal-to-noise ratio of all the computed variables for each factor. Each component (Batch, Spot or Array) are compared to the error measurement, normalized to unity for reference.

We averaged all spectra with replicates and performed all statistical analyses using nonparametric tests: the Mann-Whitney test to compare 2 groups and the Kruskal-Wallis test to compare more than 2 groups. A bootstrap method was used to perform multiple test correction in the statistical tests. The bootstrap is used to determine the probability of obtaining a particular *p*-value by chance. Group labels are randomly re-assigned (with replacement) for a total of 2,000 iterations of the bootstrap. The bootstrap method does not assume that tests are independent.

Hierarchical clustering was performed on the peak intensities of the spectra using a Spearman rank dissimilarity metric with average linkage.

## Competing interests

The author(s) declare that they have no competing interests.

## Authors' contributions

TW designed the experiments, developed the analytical approach, implemented the analysis and wrote the manuscript. DDR performed the laboratory experiments. SDV had the original idea for the study and assisted in the writing of the manuscript. All authors read and approved the manuscript.

## Supplementary Material

Additional file 1 Summary data showing stages in the quality assessment of specimen spectra. Pearson correlation coefficients were calculated for the entire spectrum prior to peak detection, the coefficient for the entire dataset is reported (Grand statistic). The coefficient of variation (CV) was calculated for peak intensities present in the entire spectrum. Click here for file
