# Supplementary material for: A method for improving SELDI-TOF mass spectrometry data quality
Source: Proteome Sci. 2007 Sep 5;5:14. doi: 10.1186/1477-5956-5-14 (PMC2040139; doi:10.1186/1477-5956-5-14)
Supplement: Additional file 1 — Summary data showing stages in the quality assessment of specimen spectra. Pearson correlation coefficients were calculated for the entire spectrum prior to peak detection, the coefficient for the entire dataset is reported (Grand statistic). The coefficient of variation (CV) was calculated for peak intensities present in the entire spectrum. [file 1477-5956-5-14-S1.pdf]

Table 1: Summary data showing stages in the quality assessment of specimen spectra.
Pearson Correlation Coefficients were calculated on the entire spectrum prior to peak detection, the coefficient for the entire dataset is reported (Grand statistic). The coefficient of variation (CV) is calculated on peak intensities present in the entire spectrum.
(A) All spectra in each specimen sample data sets
(B) Spectra remaining after passing diagnostic plot cutoff of 1-Mean r < 0.4
(C) Spectra after Partek Batch Removal.
(D) Replicate spectra averaged

Step	Data Set	Pearson Correlation Coefficient (Grand Mean ±Std Dev)	No. spectra	No. peaks	Coefficient of Variation			
					Low	High	Average	No. Peaks < 30
	CMHS_F3	0.74 ± 0.29	414	56	19.5	192.4	52.9	15
	CMHS_F4	0.82 ± 0.16	414	34	23.4	94.3	47.4	2
	CMLS_F1	0.87 ± 0.10	414	61	15.1	69.1	36.0	25
	CMLS_F3	0.76 ± 0.17	414	67	10.3	113.1	31.1	38
	CMLS_F4	0.88 ± 0.11	414	51	14.1	89.6	30.2	29
	CMLS_F5	0.80 ± 0.11	414	46	12.5	68.0	32.0	25
	CMLS_F6	0.87 ± 0.09	414	69	11.2	95.5	35.3	29
A	H50_F1	0.89 ± 0.15	414	36	16.7	118.9	55.2	4
	H50_F3	0.80 ± 0.21	414	80	10.5	214.1	33.5	51
	H50_F4	0.86 ± 0.12	414	72	9.9	121.3	35.3	36
	H50_F6	0.84 ± 0.13	414	51	16.3	158.3	42.9	13
	IMAC_F1	0.85 ± 0.11	414	59	18.8	89.2	38.8	17
	IMAC_F3	0.77 ± 0.18	414	49	16.3	119.0	43.3	12
	IMAC_F4	0.84 ± 0.15	414	45	16.1	82.3	35.1	16
	IMAC_F6	0.67 ± 0.20	414	57	14.9	85.2	40.9	17
	CMHS_F3	0.88 ± 0.08	360	55	14.2	97.6	40.6	22
	CMHS_F4	0.83 ± 0.12	403	36	23.1	94.9	45.5	2
	CMLS_F1	0.88 ± 0.08	413	61	15.0	69.2	35.5	27
	CMLS_F3	0.79 ± 0.13	389	69	9.4	107.4	29.7	41
	CMLS_F4	0.89 ± 0.08	409	51	14.2	60.2	28.9	30
	CMLS_F5	0.80 ± 0.11	414	46	12.5	68.0	32.0	25
	CMLS_F6	0.87 ± 0.08	412	69	11.2	95.4	34.6	30
B	H50_F1	0.92 ± 0.07	406	36	16.5	117.2	52.5	6
	H50_F3	0.87 ± 0.09	386	81	10.5	142.6	27.4	57
	H50_F4	0.87 ± 0.08	411	72	9.8	121.0	35.1	38
	H50_F6	0.86 ± 0.09	406	51	16.5	156.8	42.2	16
	IMAC_F1	0.86 ± 0.10	411	60	18.8	89.4	38.8	17
	IMAC_F3	0.81 ± 0.12	390	49	16.2	109.1	41.1	13
	IMAC_F4	0.86 ± 0.11	406	47	16.0	82.3	33.4	19
	IMAC_F6	0.75 ± 0.12	345	60	10.9	63.6	35.3	24
	CMHS_F3	0.93 ± 0.07	360	55	9.3	83.5	28.2	35
	CMHS_F4	0.92 ± 0.07	403	36	11.9	61.4	28.5	24
	CMLS_F1	0.92 ± 0.07	413	61	11.1	61.3	27.8	42
	CMLS_F3	0.85 ± 0.12	389	69	7.5	92.5	21.2	56

C	CMLS_F4	0.93	±	0.07	409	51	9.6	52.0	20.7	45
	CMLS_F5	0.90	±	0.08	414	46	8.9	57.3	21.7	38
	CMLS_F6	0.92	±	0.06	412	69	7.1	84.8	26.3	51
	H50_F1	0.97	±	0.03	406	36	9.8	63.8	30.8	17
	H50_F3	0.94	±	0.05	386	81	7.9	52.0	18.3	71
	H50_F4	9.30	±	0.06	411	72	7.8	75.6	24.0	55
	H50_F6	0.92	±	0.06	406	51	10.1	62.6	28.6	35
	IMAC_F1	0.90	±	0.10	411	60	14.6	78.4	28.9	38
	IMAC_F3	0.87	±	0.13	390	49	12.9	94.6	29.5	33
	IMAC_F4	0.93	±	0.07	406	47	11.2	73.8	22.3	42
	IMAC_F6	0.86	±	0.09	345	60	8.4	47.2	23.6	41
D	CMHS_F3	0.95	±	0.05	183	55	7.0	64.4	23.4	41
	CMHS_F4	0.94	±	0.06	205	36	8.8	53.3	23.5	29
	CMLS_F1	0.93	±	0.06	206	61	9.6	58.0	24.5	48
	CMLS_F3	0.88	±	0.09	203	69	6.1	83.3	18.0	64
	CMLS_F4	0.95	±	0.04	204	51	7.7	49.8	17.2	48
	CMLS_F5	0.91	±	0.07	206	46	6.7	54.4	18.4	42
	CMLS_F6	0.93	±	0.05	206	69	5.9	82.1	23.1	54
	H50_F1	0.98	±	0.02	205	36	7.4	60.8	26.1	28
	H50_F3	0.95	±	0.04	199	81	6.2	48.0	15.3	73
	H50_F4	0.94	±	0.05	206	75	5.7	67.4	20.6	60
	H50_F6	0.93	±	0.05	206	51	8.6	56.9	25.6	36
	IMAC_F1	0.92	±	0.07	205	60	11.8	74.9	24.8	45
	IMAC_F3	0.90	±	0.09	199	49	10.2	85.7	24.2	40
	IMAC_F4	0.95	±	0.04	205	47	8.5	69.8	18.1	44
	IMAC_F6	0.88	±	0.08	176	60	6.3	45.0	20.5	49
